# Opti-Med: the effectiveness of optimised clinical medication reviews in older people with ‘geriatric giants’ in general practice; study protocol of a cluster randomised controlled trial

**DOI:** 10.1186/1471-2318-14-116

**Published:** 2014-11-18

**Authors:** Floor Willeboordse, Jacqueline G Hugtenburg, Liset van Dijk, Judith E Bosmans, Oscar J de Vries, François G Schellevis, Petra J M Elders

**Affiliations:** NIVEL, Netherlands Institute for Health Services Research, Po. Box 1568, 3500 BN Utrecht, The Netherlands; Department of General Practice & Elderly Care Medicine, EMGO+Institute for Health and Care Research, VU University Medical Center, van der Boechorststraat 7, 1081 BT Amsterdam, The Netherlands; Department of Clinical Pharmacology and Pharmacy, VU University Medical Center, De Boelelaan 1117, 1081 HV Amsterdam, The Netherlands; Department of Health Sciences, EMGO Institute for Health and Care Research, Faculty of Earth and Life Sciences, Section of Health Economics & Health Technology Assessment, VU University Amsterdam, Amsterdam, The Netherlands; Department of Internal Medicine, VU University Medical Center, De Boelelaan 1117, 1081 HV Amsterdam, The Netherlands

**Keywords:** Clinical medication review, Geriatric giants, Cluster randomised controlled trial

## Abstract

**Background:**

Inappropriate drug use has been identified as one of the most important problems affecting the quality of care in older people. Inappropriate drug use may increase the risk of the occurrence of ‘geriatric giants’ such as immobility, instability, incontinence and cognitive impairment. There are indications that clinical medication reviews (CMR) can reduce inappropriate drug use. However, CMRs have not yet been implemented at a large scale in primary care. An innovative medication review program in primary care will be developed which tackles the most important obstacles for a large scale implementation of CMRs. The aim of this study is to assess whether this CMR program is (cost-) effective compared with usual general practice care for older patients with geriatric symptoms with regard to quality of life and geriatric symptoms.

**Methods:**

A cluster randomised controlled trial will be performed in 20 Dutch general practices including 500 patients. Patients of 65 years and older are eligible if they newly present with pre-specified geriatric symptoms in general practice and chronic use of at least one prescribed drug. GP practices will be stratified by practice size and randomly allocated to control (n = 10) or intervention group (n = 10). The intervention consists of CMRs which will be facilitated and prepared by an expert team consisting of a GP and a pharmacist. Primary outcome measures are patient’s quality of life and the presence of self-reported geriatric symptoms during a follow-up period of 6 months. Secondary outcomes are costs of healthcare utilisation, feasibility, number of drug related problems, medication adherence and satisfaction with medication.

**Discussion:**

This study is expected to add evidence on the (cost-) effectiveness of an optimally facilitated, prepared and structured CMR in comparison with usual care in older patients who present a geriatric symptom to their GP. The strength of this study is that it will be conducted in daily clinical practice. This improves the possibilities to implement the CMRs in the primary care setting on a large scale.

**Trial registration:**

Netherlands Trial register: NTR4264

**Electronic supplementary material:**

The online version of this article (doi:10.1186/1471-2318-14-116) contains supplementary material, which is available to authorized users.

## Background

Inappropriate drug use has been identified as one of the most important problems affecting the quality of care in older people. Appropriate drug use and prescribing in older people is difficult, because of the variability of age-related changes in the metabolism, multimorbidity and polypharmacy [1, 2]. In addition, undertreatment of especially preventive medication as well as poor treatment adherence are frequently occurring problems in older people [3]. Because of changes in pharmacokinetics and pharmacodynamics, older people are more prone to reduced effectiveness of drugs and they may be at higher risk of adverse events and other drug related problems (DRP) [4]. Several studies have shown associations between inappropriate drug use and clinical outcomes such as hospital admissions, falling, adverse drug reactions and functional decline [3, 5–8].

Inappropriate drug use may increase the risk of the occurrence and persistence of geriatric problems [3, 5, 6, 9–15]. The most common major impairments that appear in older people, also referred to as “geriatric giants” are immobility, instability, including falls and dizziness, incontinence and cognitive impairment [16]. The atypical, silent, non-specific disease presentation of a geriatric giant is a common type of symptom presentation in the older adult and associated with limitations of activities of daily living (ADL) [7]. The multi-factorial causes of geriatric giants often include DRPs which can be prescriber-related (e.g. medically non-indicated medication or inappropriate dosage), but also patient-related, e.g. ineffectiveness of drugs, adverse effects, lack of knowledge and usage of the drugs, and non-adherence [6].

There are indications that clinical medication reviews (CMR) can reduce inappropriate drug use in older people. A CMR is ‘a structured, critical examination of the patient’s medicines with the objective of reaching an agreement with the patient about treatment, optimising the impact of medicines, and minimising the number of drug related problems’ [17]. It has been shown that specific subgroups can benefit from a CMR during or immediately after hospital admissions [18, 19]. In several countries guidelines are developed for CMRs in older and polypharmacy patients [17, 20–22]. However, CMRs have not yet been implemented at a large scale because of several obstacles.

First, the evidence for the effectiveness of CMRs is not very extensive and convincing. Several studies have shown positive effects of CMRs on intermediate outcomes such as the number of DRPs, medication adherence and patient satisfaction with medication. However, these effects are heterogeneous and so far, few effects have been established on clinical outcome measures as quality of life, hospital admissions or mortality [23–26]. This lack of evidence hinders the provision of financial incentives and motivation of healthcare professionals for further implementation of CMRs.

Second, the best target group for CMRs may be unclear. At present, patients in primary care are often selected based the number of medications, the polypharmacy criteria, which is a large group and not every polypharmacy patient may need a CMR. The current study addresses the appropriateness of medication use in patients who newly present themselves with “geriatric giant” symptoms in general practice. The patients will be selected irrespective of the number of drugs used. Previous research has shown that in many of these geriatric giants, suboptimal pharmacotherapy plays an important role in the occurrence and/or persistence of the problems. Undertreatment in these patients is just as often a problem as overtreatment [27].

The third obstacle of CMR implementation is its feasibility. A CMR requires a considerable time investment for each review varying from 15 to 60 minutes for a physician and from 30 to 120 minutes for a pharmacist [28]. The current Dutch guideline for polypharmacy in older people recommends in addition to obtain the patient’s input preferably by a home visit and follows the Systematic Tool to Reduce Inappropriate Prescribing (STRIP) method, which includes at least two patient contacts [20, 29]. In addition, both lack of knowledge as well as insufficient training of both the GPs as well as the pharmacists hinder the implementation of CMRs. Finally the lack of comprehensive organisation of medical data infrastructure and exchange between professionals are hindering factors.

In this study an innovative CMR program (Opti-Med) will be developed to tackle these obstacles in a primary care setting. This Opti-Med study aims at providing scientific evidence for the effectiveness on quality of life and geriatric symptoms of an optimally facilitated, prepared and structured CMR in comparison with usual care in older patients presenting with a new geriatric giant to their GP. The feasibility of implementing the program in the daily routine of several GP practices will be evaluated.

## Methods

The Opti-Med study protocol was approved by the Medical Ethics Committee of the VU University Medical Centre Amsterdam (reference 2011/408). For the description of the design of the Opti-Med intervention, the Consolidated Standards of Reporting Trials (CONSORT) statement with extension to cluster randomised trials is followed [30].

### Study design

**Figure 1:**
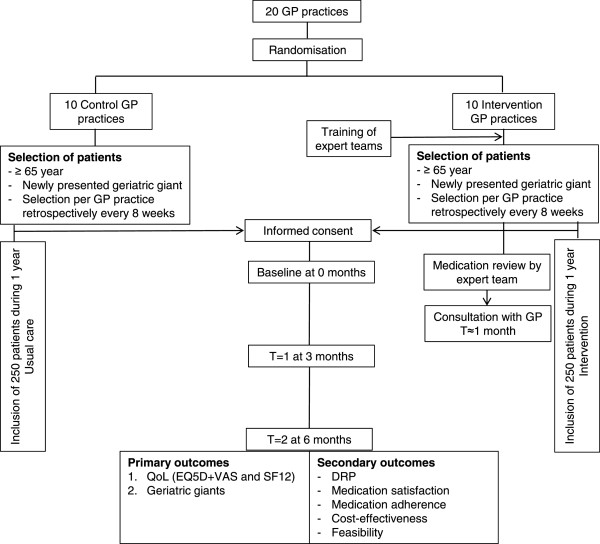
**Study design of Opti**-**Med.**

A cluster randomised clinical trial will be performed in 20 general practices including 500 patients (see Figure 1). Allocation of the intervention and control condition will be carried out randomly at practice level. Eligible patients will be invited to participate in the study and the medication of the patients listed in the intervention practices will be reviewed. Patients listed in the control practices will receive usual care, with no systematic attention for their medication. The effects of this intervention will be assessed after a follow-up period of 6 months. The rationale to use a cluster design at practice level is to prevent contamination of structural attention to CMR activities within the GP practice.

### Setting

The study will be embedded in the Academic Network of General Practices of the VU University Medical Centre (ANH-VUmc) that consists of 20 GP practices in Amsterdam, the Netherlands. Similar to almost all GPs in the Netherlands, the GPs in this network use electronic medical record systems in which all patients contact diagnoses are coded using the International Classification of Primary Care first edition (ICPC-1) [31]. All practices employ practice nurses who can assist with the implementation of the intervention, have not yet systematically implemented structured CMR and are therefore eligible for this trial.

This CMR program is tuned to the Dutch healthcare setting where patients are listed with a general practice and their GP is the first contact point for a patient with healthcare problems. Only in case of emergency or after referral by the GP, patients visit secondary care professionals. Moreover, in the Netherlands it is common to visit one main pharmacy which provides all prescription medication. As such, the pharmacist has the most accurate and complete medication data.

### Randomisation

Randomisation will be performed by a statistician blinded to characteristics of the practices using a computer generated list of random numbers. The practices are stratified by practice size (two strata), to ensure equally sized groups. Before patients are recruited, participating practices will be randomly allocated to the intervention, or control condition. Patients will be allocated to either one of the treatment conditions, based on the practice where they are listed. Blinding of patients, GPs and practice nurses to treatment allocation is not possible due to the nature of the intervention.

### Participants

Patients of 65 years and older are eligible if they newly present with a geriatric giant in general practice use at least one prescription drug chronically. A new geriatric giant is defined as being a first episode if the problem has not been noted in the patients’ medical file during the previous 12 months. Patients with geriatric giants are identified on the basis of the ICPC coded diagnoses [31] (see Additional file 1) in their electronic medical record. Chronic use of at least one drug is defined as at least three prescriptions in the last 12 months in the GP practice. In The Netherlands, prescriptions are always for a maximum of three months of treatment.

### Screening questionnaire

Together with the invitation for participation in the study, both intervention and control patients receive a screening questionnaire. The questionnaire consists of four parts:

I. Questions on the presence and self-perceived severity of geriatric giants using visual analogue scales (VAS) (0–10).The geriatric problems that are evaluated are 1. Mobility problem; 2. Dizziness; 3. Urinary incontinence; 4. Problems with cognition; 5. Fear of Falling [32]; Also a question is formulated regarding the number of falls in the previous 6 months;
II. Questions regarding body weight (kg), length (m) and pain (VAS 0–10);
III. Actual drug use including OTC drugs;
IV. Questionnaire aimed at the identification of DRPs from the patient perspective.

Part III and IV of the questionnaire are developed by the authors of this study and have been shown to have good agreement with a patient interview during a home visit [33, 34].

### Inclusion procedure

For identifying potential participants in the practices a two-step approach will be applied.

- Step 1. Eligible patients will be identified retrospectively every 8 weeks on the basis of a selected set of ICPC codes (see Additional file 1), age and chronic use of at least one drug, through a predefined search strategy in the GP electronic medical records. Only patients who consulted the GP with one of these diagnoses in this time period and who did not present with this problem to the GP during the previous 12 months are eligible to participate.
- Step 2. Identified potential participants in step 1 will be invited to fill out the above described screening questionnaire. If needed, the patient will be offered support at home to fill out the screening questionnaire. Patients will be included in the trial if they indicate to currently have a score of five or higher on one of the VAS scales of the geriatric problems or have indicated to have one fall or more in the previous 6 months. Additionally they have to indicate that they are willing to participate in the trial by signing an informed consent form.

#### Exclusion criteria

Patients are not eligible when they have a diagnosis of dementia in their medical record. In addition, patients with a Mini Mental State Examination (MMSE) score of 18 or less will be excluded from the trial, since this is the cut off for serious cognitive impairment [35, 36]. An MMSE interview is only carried out when patients indicated they needed help to fill out the screening questionnaire. Each 8 weeks, the GPs will receive a list of all eligible patients and they will exclude patients who received a structured CMR in the last 6 months or are according to the GP unable to participate (e.g. due to terminal illness or severe psychiatric problems).

### Intervention

#### Preparatory steps

The research assistant prepares together with the practice nurse the CMRs for the expert teams (see below). The required information from the electronic medical files from the GP practice, the pharmacy and the screening questionnaire is collected. This information consists of the actual drug use of the patient including OTC drugs, drug delivery history, potential DRPs, the medical problems of the patient, laboratory test results, e.g. renal function and other measurements such as blood pressure.

#### Clinical medication review by expert team

An expert team consisting of a GP (not the patient’s GP) and a pharmacist (not the patient’s pharmacist) will review the medication. The team will carry out a systematic assessment aimed at identifying drug related problems (DRP) experienced by the patient as indicated in the screening questionnaire and at optimising the medication of the patient. The medication will be structurally reviewed according to the Dutch Systematic Tool to Reduce Inappropriate Prescribing (STRIP) method including the translated Screening Tool of Older Person’s Prescriptions (STOPP) and Screening Tool to Alert doctors to Right Treatment (START) criteria [20, 29]. A computer assisted version of the STRIP method, the STRIP-assistant will be used by the team [37]. First, the medications are linked to the diseases or symptoms. Then the following steps will be systematically followed:

1. Undertreatment
2. Effectiveness of treatment
3. Overtreatment
4. Potential adverse events
5. Interactions and contra-indications
6. Dosing problems
7. Other problems, such as user problems, knowledge or adherence

Finally, the result of this CMR analysis is a pharmacotherapeutic treatment plan that will be sent to the patient’s GP (see Additional file 2) Three expert teams will be formed for the Opti-Med study.

#### Implementing the pharmacotherapeutic treatment plan

Patients will be invited for a consultation with the GP in which both the pharmacotherapeutic treatment plan and the patient’s perspective as previously assessed with the screening questionnaire are discussed and definitive changes in the prescribed medication will be implemented.

#### Monitoring the medication use

Six months after inclusion a check will be carried out by the researchers to identify medication changes compared to the outcome of the CMR (intervention group) and/or compared to baseline drug use of all participating patients. The patients’ GPs will receive a signal if new DRPs are identified. The follow-up of this signal is outside the scope of this study.

#### Training

Expert teams will follow accredited online courses for medication reviews and pharmacology in elderly and two medication review workshops. Participating GPs and practice nurses of the intervention practices will be instructed how to carry out the study protocol and will receive a handbook.

### Control condition

Eligible patients who are listed in a control practice will be identified and selected in exactly the same manner as in the intervention group. They will be asked to give informed consent and to fill in the same questionnaires at inclusion, baseline, 3 and 6 months. Patients in control practices receive usual care, which means that no structured attention will be paid to their medication.

### Outcome measures

Measurements by means of patient questionnaires and proxy assessments will be carried out at baseline, after 3 and 6 months It is expected that some patients will develop either cognitive problems or other difficulties precluding that they fill out the questionnaires adequately during the study. Self-assessment in these patients might therefore be less reliable or become not feasible. Proxy assessment could be a substitute for self-assessment of quality of life [38]. Therefore, patients will be asked to indicate two proxies: an informal care giver and a professional care giver who will fill out a proxy assessment questionnaire of the patient’s quality of life. Data on morbidity and laboratory test results will be collected using medical records in the GP practices. Characteristics of medication, changes in medication and adherence to medication will be assessed using dispensing data from the patient’s pharmacist. All outcomes will be assessed at patient level.

### Primary outcome measures

Quality of life (QoL) will be assessed using both the SF-12 and the EuroQol (EQ-5D-3 L) at baseline, and after 3 and 6 months. The SF-12 covers eight dimensions of health with two summary scores; physical health (PHS) and mental health (MHS), and has been validated in many different countries and populations [39–42]. The PHS will be used as outcome measure because of its superior responsiveness compared to the MHS [43]. The EQ-5D-3 L is a generic preference based health status measure that has been shown to be valid and reliable in a variety of populations and patient groups [44, 45]. The EQ-5D-3 L will be assessed using information from the patient and by proxy assessment by an informal carer and a healthcare professional. The proxies will be asked to report on QoL from the patients’ perspective [46]. The EQ-5D scores will be used to calculate utilities using the Dutch tariffs. Quality-adjusted life years (QALYs) will be calculated using linear interpolation between time points. Higher QALY scores indicate more improvement in quality of life [47].

The presence of geriatric giants will be assessed at baseline with the screening questionnaire. At 3 and 6 months, presence of geriatric giants will be assessed with the same questions as in the screening questionnaire enabling to assess changes compared to baseline.

### Secondary outcome measures

The prevalence of DRPs in patients will be determined at baseline and after 6 months in both groups. The 6 month questionnaire will be similar to the screening questionnaire omitting questions that only need to be asked at the beginning of the study. An independent clinical pharmacologist and GP will assess the DRPs on the basis of the screening questionnaire and the pharmacist’s medication overview using the DOCUMENT checklist [48]. DOCUMENT stands for Drug selection, Over- or underdose prescribed, Compliance, Untreated indications, Monitoring, Education or information, Non-clinical and Toxicity or adverse reaction and has multiple subcategories.

Research suggests that greater treatment satisfaction is associated with better compliance [49]. Patient satisfaction about medication in general will be assessed by the single-item Medication Satisfaction Questionnaire (MSQ) “Overall, how satisfied or dissatisfied are you with your current medication?” with a written response assessed on a seven point Likert like scale at baseline, 3 and 6 months [50].

Medication adherence will be measured in two ways; 1. Check for at least one pharmacy delivery in the last six months for all chronically used medication and 2. Self-reported adherence as questioned in the screening and follow-up questionnaires at baseline and 6 months. Self-reported adherence is part of the developed screening questionnaire.

Costs will be measured from a societal perspective. To calculate the costs of the intervention, information will be recorded by the expert team, the GP, the pharmacist and the practice nurse in terms of time and material spent on performing the CMR and the monitoring of the patients. Healthcare costs made by the patient will be assessed from a societal perspective using an slightly adapted version of the Dutch Medical Consumption Questionnaire (*i*MTA) questionnaire on care consumption, including informal care after 3 months (t = 1) and after 6 months [51]. Information on prescribed medication will be derived from the pharmacy administration information system (PAIS). Lost productivity costs will not be included since almost all patients will be retired. Healthcare utilisation will be valued according to guidelines for economic evaluation in healthcare in the Netherlands [52].

### Pilot study

Based on the experiences of two small pilot innovation programs, the intervention program was developed ([53] and unpublished results Elders and Bleeker 2009). An Opti-Med pilot study was conducted in two intervention and two control practices for eight weeks including 10 patients. An evaluation was conducted to test the logistics, baseline measurements, questionnaires, and the feasibility and functioning of the expert team of the study. The pilot study resulted in minor changes in the questionnaire instructions, improvements in logistics and communication with the GP practices.

### Process analysis

The process evaluation involves assessing the extent to which the intervention is performed according to the protocol of the study, the time that is spent by the professionals to perform the activities of the protocol, the nature of the recommendations made to the patients by the GP, compliance with these recommendations, the judgment of the GPs, pharmacists and practice nurses about the intervention program. Data on these topics are collected using structured registration forms during the intervention. In addition, semi-structured interviews will be held with the participating practice nurses, GPs and members of the expert teams at the end of the intervention period in order to record their experiences and opinions on the CMR program. The presence and influence of possible contamination in both intervention and control practices will also be assessed by interviewing GPs or practice nurses at the end of the trial on their opinions to what degree structured attention to medication was an issue during the study period in general or with regard to specific patients.

### Sample size

The size of the study groups is based on the difference in change over 6 months between the intervention and control group of the EQ-5D VAS score (score range 0–100). A difference of 7.4 in the EQ-5D VAS is considered as a clinically relevant difference [54]. The average score among persons with osteoarthritis, a comparable group, is 64.8 (standard deviation 26.5). To establish a difference of 7.4 points as statistically significant with alpha = 0.05 and beta = 0.20, a group size of 225 is sufficient, taking the clustered design into account. To adjust for loss to follow up of 10% we will include 500 patients.

### Statistical analyses

Descriptive statistics will be used to describe the study population. Dropout and loss to follow up will be described. Effect analyses will be performed according to both ‘intention to treat’ and per protocol principles. Differences between intervention and usual care patients on the outcome measures will be compared between the intervention and control group by both univariate and multivariate techniques. Multilevel linear and logistic regression analyses will be performed to study differences between the intervention and the control patients. Multilevel analysis is needed in order to take clustering on the GP level and repeated measurements in one patient into account. We will adjust for possible confounders, such as gender, age, education level, number of medications and multi-morbidity.

Possible future subgroup analyses will be exploratory, due to lack of power.

### Economic evaluation

For the economic evaluation, missing cost and effect data will be imputed using multiple imputation according to the Multivariate Imputation by Chained Equations (MICE) algorithm developed by van Buuren using predictive mean matching and fully conditional specification [55]. The number of imputed datasets will be increased until the fraction of missing information is below 5% [56]. The imputed datasets will be analysed separately as described below and subsequently pooled using Rubin’s rules.

The effect measures that will be taken into account in the cost-effectiveness are QALYs, and changes in the VAS scores of the geriatric giant symptoms. For effects and costs, linear multilevel regression models will be estimated. Clustering at the level of GP practice will be included in these multilevel models. Incremental cost-effectiveness ratios (ICERs) will be calculated by dividing the difference in mean total costs between the treatment and control groups by the difference in mean effects between the groups. Costs generally have a highly skewed distribution; therefore, bootstrapping with 5,000 replications will be used to estimate bias-corrected and accelerated confidence intervals around cost differences [57]. To account for the clustering of data, bootstrap replications will be stratified for practice [58]. The uncertainty surrounding the ICERs which will be graphically presented on cost-effectiveness planes. Cost-effectiveness acceptability curves and net monetary benefits will also be calculated. Cost-effectiveness acceptability curves show the probability that the medication review programme is cost-effective in comparison with usual care for a range of different ceiling ratios thereby showing decision uncertainty.

## Discussion

This study is expected to add evidence on the effectiveness and cost-effectiveness of an optimally facilitated, prepared and structured CMR in comparison with usual care in older patients presenting with a geriatric giant to their GP.

Geriatric giants are highly prevalent among older people and represent a major cause of impaired quality of life. Optimising the patient’s medication in addition to treating these geriatric problems or delaying their worsening by treatment, is expected to have a positive additional impact on the patient’s perceived health. Moreover, optimising drug use will also improve the effectiveness of drug treatment, prevent adverse drug reactions and potentially harmful drug interactions, and consequently hospital admissions or even death.

For healthcare professionals, handling DRPs in older people is a challenge. The burden of aging on the healthcare sector, care efficiency is an important issue.

The streamlining of the process, the experienced expert teams and minimising the contact moments with the patients due to the written questionnaire as proposed in this study increases the feasibility that CMRs can be implemented successfully in usual care. In practice, after this study, expert teams could be implemented in a GP cooperation or another regional care settings in which pharmacists and GPs should be trained and form expert groups.

The strength of this study is that it will be conducted in daily clinical practice and will resemble daily clinical practice as much as possible. This improves the possibilities to implement CMRs in the primary care setting.

The most important innovations compared to previous programs are:

1. The CMR will not focus primarily on polypharmacy patients, instead the selection of older patients is based on episodes related to a geriatric giant;
2. The CMR is prepared by a trained expert team consisting of an external GP and an external pharmacist who formulate a pharmacotherapeutic treatment plan for the patient’s GP;
3. The coordination of the CMR at the primary care level is performed by a case manager, usually the practice nurse or assistant;
4. The number of contacts with the patient is reduced by assessing the patient’s perspective by a written questionnaire instead of a home visit.

Quality of life is the primary outcome measure. This may not be sensitive enough to capture the changes induced by the intervention. However, we have chosen to use generic health measures as primary outcome measures because we include patients in this study that might have a variety of geriatric symptoms with a heterogeneous treatment effect. Also we think that in this population quality of life is the most important outcome.

In addition, the study is not blinded and there is a possibility of contamination between the intervention and control group. We counter possible contamination between treatments groups by using a cluster randomised controlled design. That way, caregivers cannot unintentionally apply aspects of the Opti-Med study into their usual care for patients. Patients in the control condition also fill out the screening questionnaire on actual drug use and medication related problems, this is needed for the study, but could underestimate the effectiveness of Opti-Med by increasing awareness of possible drug related problems in these control participants. Furthermore, we suppose that current activities on older people care and possible future pharmacist’s polypharmacy projects are minimally interfering with the study. This assumption about possible contamination will be checked during the process analysis.

Implementation of structured CMR and monitoring will raise the awareness about the importance of optimising medication use in general, and especially in older patients among healthcare professionals. In this study, we will evaluate a form of a structured CMR that can easily be implemented in a GP cooperation or care group. If the CMR is shown (cost-) effective and feasible it could also be extrapolated to other groups of patients in the future in whom inappropriate medication use is suspected as well. If proven cost-effective, this will support the nationwide implementation of this structured approach. The first results of the study will be expected at the end of 2015.

## Electronic supplementary material

Additional file 1: **ICPC codes for the selection of patients with geriatric giants in the Opti-Med study.** (PDF 22 KB)

Additional file 2: **Format Pharmacotherapeutic Treatment Plan Opti-Med.** (PDF 102 KB)

## Abbreviations

ADL: Activities of daily living
CMR: Clinical medication review
CONSORT: Consolidated standards of reporting trials
DRP: Drug related problems
GP: General practitioner
ICER: Incremental cost-effectiveness ratio
ICPC: International classification of primary care
MICE: Multivariate imputation by Chained Equations
MMSE: Mini mental state examination
OTC: Over the counter
STRIP: Systematic tool to reduce inappropriate prescribing
PAIS: Pharmacy administration information system
QoL: Quality of life
QALY: Quality-adjusted life year
STOPP: Screening tool of older person’s prescriptions
START: Screening tool to alert doctors to right treatment
VAS: Visual analogue scale.

## References

[1] Mallet L, Spinewine A, Huang A (2007). The challenge of managing drug interactions in elderly people. Lancet.

[2] Spinewine A, Schmader KE, Barber N, Hughes C, Lapane KL, Swine C, Hanlon JT (2007). Appropriate prescribing in elderly people: how well can it be measured and optimised?. Lancet.

[3] Leendertse AJ, Egberts AC, Stoker LJ, van den Bemt PM (2008). Frequency of and risk factors for preventable medication-related hospital admissions in the Netherlands. Arch Intern Med.

[4] Jansen PAF, Brouwers JABJ (2012). Clinical pharmacology in older persons. Scientifica.

[5] Berdot S, Bertrand M, Dartigues JF, Fourrier A, Tavernier B, Ritchie K, Alperovitch A (2009). Inappropriate medication use and risk of falls–a prospective study in a large community-dwelling elderly cohort. BMC Geriatr.

[6] Hanlon JT, Fillenbaum GG, Kuchibhatla M, Artz MB, Boult C, Gross CR, Garrard J, Schmader KE (2002). Impact of inappropriate drug use on mortality and functional status in representative community dwelling elders. Med Care.

[7] Jarrett PG, Rockwood K, Carver D, Stolee P, Cosway S (1995). Illness presentation in elderly patients. Arch Intern Med.

[8] Rogers S, Wilson D, Wan S, Griffin M, Rai G, Farrell J (2009). Medication-related admissions in older people: a cross-sectional, observational study. Drugs Aging.

[9] French L, Phelps K, Pothula NR, Mushkbar S (2009). Urinary problems in women. Prim Care.

[10] Gerretsen P, Pollock BG (2011). Drugs with anticholinergic properties: a current perspective on use and safety. Expert Opin Drug Saf.

[11] Leon C, Gerretsen P, Uchida H, Suzuki T, Rajji T, Mamo DC (2010). Sensitivity to antipsychotic drugs in older adults. Curr Psychiatry Rep.

[12] Roughead EE, Semple SJ (2009). Medication safety in acute care in Australia: where are we now? Part 1: a review of the extent and causes of medication problems 2002–2008. Aust New Zealand Health Policy.

[13] Woolcott JC, Richardson KJ, Wiens MO, Patel B, Marin J, Khan KM, Marra CA (2009). Meta-analysis of the impact of 9 medication classes on falls in elderly persons. Arch Intern Med.

[14] Unwin BK, Porvaznik M, Spoelhof GD (2010). Nursing home care: part II. Clinical aspects. Am Fam Physician.

[15] Chrischilles E, Rubenstein L, Van Gilder R, Voelker M, Wright K, Wallace R (2007). Risk factors for adverse drug events in older adults with mobility limitations in the community setting. J Am Geriatr Soc.

[16] Isaacs B (1965). An Introduction to Geriatrics.

[17] Task Force on Medicines Partnership and The National Collaborative Medicines Management Services Programme (2002). Room for Review: a Guide to Medication Review: the Agenda for Patients, Practitioners and Managers.

[18] Gillespie U, Alassaad A, Henrohn D, Garmo H, Hammarlund-Udenaes M, Toss H, Kettis-Lindblad A, Melhus H, Morlin C (2009). A comprehensive pharmacist intervention to reduce morbidity in patients 80 years or older: a randomized controlled trial. Arch Intern Med.

[19] Murray MD, Young J, Hoke S, Tu W, Weiner M, Morrow D, Stroupe KT, Wu J, Clark D, Smith F, Gradus-Pizlo I, Weinberger M, Brater DC (2007). Pharmacist intervention to improve medication adherence in heart failure: a randomized trial. Ann Intern Med.

[20] Nederlands Huisarts Genootschap (NHG) (2012). Multidisciplinaire richtlijn Polyfarmacie bij ouderen.

[21] Pharmaceutical Society of Australia Ltd (2011). Guidelines for Pharmacists Providing Home Medicines Review (HMR) Service.

[22] Clyne WB, Blenkinsopp A, Seal R (2008). A Guide to Medication Review.

[23] Patterson SM, Hughes C, Kerse N, Cardwell CR, Bradley MC (2012). Interventions to improve the appropriate use of polypharmacy for older people. Cochrane Database Syst Rev.

[24] Holland R, Desborough J, Goodyer L, Hall S, Wright D, Loke YK (2008). Does pharmacist-led medication review help to reduce hospital admissions and deaths in older people? A systematic review and meta-analysis. Br J Clin Pharmacol.

[25] Nkansah N, Mostovetsky O, Yu C, Chheng T, Beney J, Bond CM, Bero L (2010). Effect of outpatient pharmacists’ non-dispensing roles on patient outcomes and prescribing patterns. Cochrane Database Syst Rev.

[26] Lehnbom EC, Stewart MJ, Manias E, Westbrook JI (2014). Impact of medication reconciliation and review on clinical outcomes. Ann Pharmacother.

[27] Frankfort SV, Tulner LR, van Campen JP, Koks CH, Beijnen JH (2006). Evaluation of pharmacotherapy in geriatric patients after performing complete geriatric assessment at a diagnostic day clinic. Clin Drug Investig.

[28] Mast RM, Schouten GP, van Woerkom M (2010). Niveau van medicatiebooordeling initiatieven in Nederland kan beter. Pharmaceutisch Weekblad.

[29] Vermeulen Windsant-van den Tweel AMA, Verduijn MM, Derijks HJ, van Marum RJ (2012). Detectie van ongeschikt medicatiegebruik bij ouderen: worden de STOPP- en START criteria de nieuwe standaard?. Ned Tijdschr Geneeskd.

[30] Campbell MK, Piaggio G, Elbourne DR, Altman DG (2012). Consort 2010 statement: extension to cluster randomised trials. BMJ.

[31] Lamberts HWM (1987). ICPC International Classification of Primary Care.

[32] Scheffer AC, Schuurmans MJ, van Dijk N, van der Hooft T, de Rooij SE (2010). Reliability and validity of the visual analogue scale for fear of falling in older persons. J Am Geriatr Soc.

[33] Ahmad A, Mast MR, Nijpels G, Elders PJ, Dekker JM, Hugtenburg JG (2014). Identification of drug-related problems of elderly patients discharged from hospital. Patient Prefer Adherence.

[34] Willeboordse F, Grundeken LH, van den Eijkel LP, Elders PJ, Hugtenburg JG, Schellevis FG (2014). Patiënt vragenlijst over medicatiegebruik en geneesmiddel gerelateerde problemen vanuit het patiëntenperspectief.

[35] Wind AW, Gussekloo J, Vernooij-Dassen MJGJ, Bouma M, Boomsma LJ, Boukes FS (2003). NHG-standaard dementie: tweede herziening. Huisarts Wet.

[36] Folstein MF, Folstein SE, McHugh PR (1975). Mini-mental state. A practical method for grading the cognitive state of patients for the clinician. J Psychiatr Res.

[37] Meulendijk M, Spruit M, Drenth-van MC, Numans M, Brinkkemper S, Jansen P (2013). General practitioners’ attitudes towards decision-supported prescribing: an analysis of the Dutch primary care sector. Health Informatics J.

[38] McPhail S, Beller E, Haines T (2008). Two perspectives of proxy reporting of health-related quality of life using the Euroqol-5D, an investigation of agreement. Med Care.

[39] Aaronson NK, Muller M, Cohen PD, Essink-Bot ML, Fekkes M, Sanderman R, Sprangers MA, Te VA, Verrips E (1998). Translation, validation, and norming of the Dutch language version of the SF-36 Health Survey in community and chronic disease populations. J Clin Epidemiol.

[40] Brazier J, Jones N, Kind P (1993). Testing the validity of the Euroqol and comparing it with the SF-36 health survey questionnaire. Qual Life Res.

[41] Gandek B, Ware JE, Aaronson NK, Apolone G, Bjorner JB, Brazier JE, Bullinger M, Kaasa S, Leplege A, Prieto L, Sullivan M (1998). Cross-validation of item selection and scoring for the SF-12 Health Survey in nine countries: results from the IQOLA Project. International quality of life assessment. J Clin Epidemiol.

[42] Ware J, Kosinski M, Keller SD (1996). A 12-item short-form health survey: construction of scales and preliminary tests of reliability and validity. Med Care.

[43] Haywood KL, Garratt AM, Fitzpatrick R (2005). Older people specific health status and quality of life: a structured review of self-assessed instruments. J Eval Clin Pract.

[44] The EuroQoL Group (1990). EuroQol–a new facility for the measurement of health-related quality of life. The EuroQol Group. Health Policy.

[45] Brooks R (1996). EuroQol: the current state of play. Health Policy.

[46] Pickard AS, Knight SJ (2005). Proxy evaluation of health-related quality of life: a conceptual framework for understanding multiple proxy perspectives. Med Care.

[47] Lamers LM, Stalmeier PF, McDonnell J, Krabbe PF, van Busschbach JJ (2005). Measuring the quality of life in economic evaluations: the Dutch EQ-5D tariff. Ned Tijdschr Geneeskd.

[48] Williams M, Peterson GM, Tenni PC, Bindoff IK, Stafford AC (2012). DOCUMENT: a system for classifying drug-related problems in community pharmacy. Int J Clin Pharm.

[49] Barbosa CD, Balp MM, Kulich K, Germain N, Rofail D (2012). A literature review to explore the link between treatment satisfaction and adherence, compliance, and persistence. Patient Prefer Adherence.

[50] Vernon MK, Revicki DA, Awad AG, Dirani R, Panish J, Canuso CM, Grinspan A, Mannix S, Kalali AH (2010). Psychometric evaluation of the Medication Satisfaction Questionnaire (MSQ) to assess satisfaction with antipsychotic medication among schizophrenia patients. Schizophr Res.

[51] Bouwmans C, Hakkaart-van Roijen L, Koopmanschap M, Krol M, Severens H, Brouwer W (2013). Medical Cost Questionnaire (iMCQ).

[52] Hakkaart-van Roijen L, Tan SS, Bouwmans CAW (2010). Handleiding voor kostenonderzoek: methoden en standaard kostprijzen voor economische evaluaties in de gezondheidszorg. College voor Zorgverzekeringen Den Haag The Netherlands.

[53] Ahmad A, Hugtenburg J, Welschen LM, Dekker JM, Nijpels G (2010). Effect of medication review and cognitive behaviour treatment by community pharmacists of patients discharged from the hospital on drug related problems and compliance: design of a randomized controlled trial. BMC Public Health.

[54] Walters SJ, Brazier JE (2005). Comparison of the minimally important difference for two health state utility measures: EQ-5D and SF-6D. Qual Life Res Int J Qual Life Asp Treat Care Rehab.

[55] Van Buuren S (2007). Multiple imputation of discrete and continuous data by fully conditional specification. Stat Methods Med Res.

[56] White IR, Royston P, Wood AM (2011). Multiple imputation using chained equations: Issues and guidance for practice. Stat Med.

[57] Joling KJ, Bosmans JE, van Marwijk HW, van der Horst HE, Scheltens P, Vroomen JL, van Hout HP (2013). The cost-effectiveness of a family meetings intervention to prevent depression and anxiety in family caregivers of patients with dementia: a randomized trial. Trials.

[58] van der Leeden R (2008). Handbook of Multilevel Analysis: Resampling Mulitlevel Models.

[CR59] The pre-publication history for this paper can be accessed here:http://www.biomedcentral.com/1471-2318/14/116/prepub

