# Supplementary material for: Opti-Med: the effectiveness of optimised clinical medication reviews in older people with ‘geriatric giants’ in general practice; study protocol of a cluster randomised controlled trial
Source: BMC Geriatr. 2014 Nov 18;14:116. doi: 10.1186/1471-2318-14-116 (PMC4240827; doi:10.1186/1471-2318-14-116)
Supplement: Supplementary file 1 — Additional file 1: ICPC codes for the selection of patients with geriatric giants in the Opti-Med study. (PDF 22 KB) [file 12877_2014_1051_MOESM1_ESM.pdf]

Additional file 1. ICPC codes for the selection patients with geriatric giants in the Opti-Med study

Category	Code	Description
General	A05	Feeling ill
Instability / immobility	A06	Fainting/syncope
	A10	Bleeding-haemorrhage not otherwise specified
	A28	Limited function/disability not otherwise specified
	A80	Trauma/injury not otherwise specified
	H82	Vertiginous syndrome / labyrinthitis
	K88	Postural hypotension
	L02	Back symptom/complaint
	L03	Low back symptom/complaint without radiating pain
	L13	Hip symptom/complaint
	L14	Leg/thigh symptom/complaint
	L15	Knee symptom/complaint
	L16	Ankle symptom/complaint
	L17	Foot/toe symptom/complaint
	L28	Limited function/disability
	L72	Fracture: radius/ulna
	L73	Fracture: tibia/fibula
	L74	Fracture: hand/foot bone
	L75	Fracture: femur
	L76	Fracture: other
	L77	Sprain/strain of ankle
	L78	Sprain/strain of knee
	L79	Sprain/strain of joint not otherwise specified
	L80	Dislocation/subluxation
	L81	Injury musculoskeletal not otherwise specified
	L86	Low back symptom/complaint with radiating pain
	L96	Acute internal damage knee
	N17	Vertigo/dizziness
	N18	Paralysis/weakness
	N79	Concussion
	N80	Head injury other
	S16	Bruise/contusion
	S17	Abrasion/scratch/blister
	S18	Laceration/cut
	S19	Skin injury other
Cognitive impairment	P20	Memory / concentration / orientation disturbance
	P71	Organic psychosis other
	P73	Affective psychosis
	P01	Feeling anxious/nervous/tense
	P03	Feeling depressed
	P05	Senility, feeling/behaving old
	P74	Anxiety disorder/anxiety state
	P76	Depressive disorder
Urine incontinence	U04	Incontinence urine
