# Supplementary material for: Opti-Med: the effectiveness of optimised clinical medication reviews in older people with ‘geriatric giants’ in general practice; study protocol of a cluster randomised controlled trial
Source: BMC Geriatr. 2014 Nov 18;14:116. doi: 10.1186/1471-2318-14-116 (PMC4240827; doi:10.1186/1471-2318-14-116)
Supplement: Supplementary file 2 — Additional file 2: Format Pharmacotherapeutic Treatment Plan Opti-Med. (PDF 102 KB) [file 12877_2014_1051_MOESM2_ESM.pdf]

Additional file 2. Format Pharmacotherapeutic Treatment Plan Opti-Med

**Pharmacotherapeutic Treatment Plan Opti-Med**	
Date consultation	
Name GP	
Time investment (incl. preparation)	Minutes | Home visit: YES / NO

**Name patiënt:**	**Date of birth:**	**Follow-up number (Opti-Med):**
**Medicationreview date:**	**Medication review executed by team no:**	**GP practice:**
**Geriatric giant(s):**		
**Diseases and symptoms based on ICPC**	**New medication list** ***(new items are bold)***	**Problems/questions/signals from patient:**

**Priority**	**No**	**Proposed changes/advices**	**Explanation / reason:**	**Follow up? Yes/No/NA + explain:**
		**Start:**		YES/NO/NA
		**Stop:**		YES/NO/NA
		**Changes (frequency, dosing):**		YES/NO/NA
		**Ask/discuss with patient:**		YES/NO/NA
		**Additional laboratory results/physical assessment:**		YES/NO/NA
		**Other advice:**		YES/NO/NA

Other changes (related to the medication regime) by GP?

FINALLY: - Communicate all changes with the pharmacy
- When needed, provide the patient with written information on changes in medication
